# Proterozoic Acquisition of Archaeal Genes for Extracellular Electron Transfer: A Metabolic Adaptation of Aerobic Ammonia-Oxidizing Bacteria to Oxygen Limitation

**DOI:** 10.1093/molbev/msad161

**Published:** 2023-07-13

**Authors:** Arda Gulay, Greg Fournier, Barth F Smets, Peter R Girguis

**Affiliations:** Department of Organismic and Evolutionary Biology, Harvard University, Cambridge, MA, USA; Department of Environmental and Resource Engineering, Technical University of Denmark, Lyngby, Denmark; Department of Earth, Atmospheric and Planetary Sciences, Massachusetts Institute of Technology, Cambridge, MA, USA; Department of Environmental and Resource Engineering, Technical University of Denmark, Lyngby, Denmark; Department of Organismic and Evolutionary Biology, Harvard University, Cambridge, MA, USA

**Keywords:** extracellular electron transfer, ammonia oxidizing bacteria, multiheme cytochromes, horizontal gene transfer, hypoxic

## Abstract

Many aerobic microbes can utilize alternative electron acceptors under oxygen-limited conditions. In some cases, this is mediated by extracellular electron transfer (or EET), wherein electrons are transferred to extracellular oxidants such as iron oxide and manganese oxide minerals. Here, we show that an ammonia-oxidizer previously known to be strictly aerobic, *Nitrosomonas communis*, may have been able to utilize a poised electrode to maintain metabolic activity in anoxic conditions. The presence and activity of multiheme cytochromes in *N. communis* further suggest a capacity for EET. Molecular clock analysis shows that the ancestors of *β-*proteobacterial ammonia oxidizers appeared after Earth's atmospheric oxygenation when the oxygen levels were >10^−4^*p*O_2_ (present atmospheric level [PAL]), consistent with aerobic origins. Equally important, phylogenetic reconciliations of gene and species trees show that the multiheme c-type EET proteins in *Nitrosomonas* and *Nitrosospira* lineages were likely acquired by gene transfer from γ*-*proteobacteria when the oxygen levels were between 0.1 and 1 *p*O_2_ (PAL). These results suggest that *β-*proteobacterial EET evolved during the Proterozoic when oxygen limitation was widespread, but oxidized minerals were abundant.

**Significance**

Metabolic versatility can permit typically aerobic microbes to survive under conditions when oxygen is unavailable as a terminal electron acceptor. The presented work demonstrates a previously unidentified anaerobic extracellular electron transfer metabolism that operates in aerobic *β-*proteobacterial ammonia oxidizers and reconstructs the evolutionary history of this metabolism, linking it to the history of Earth's oxygenation. Our approach contributes to understanding metabolisms in the N-cycle and their evolution on Earth, as well as how aerobic microbes manage to survive under oxygen-limited or -depleted conditions.

## Introduction

Many microbes employ diverse energy metabolisms that ensure their survival across a range of environmental conditions, for example utilizing different electron donors and/or acceptors for redox couples (Berney et al. 2014; Koch et al. 2015; Bayer et al. 2021). One physiological capacity that enables this flexibility is extracellular electron transfer (EET), which involves transferring electrons to exogenous materials outside the cell rather than soluble electron acceptors within the cell (Shi et al. 2016). EET is enabled through a diversity of mechanisms, including redox-active soluble compounds as well as multiheme c-type cytochromes (MHCs), which are anchor proteins that link intracellular energy pathways to redox transformations of extracellular metal ions (McGlynn et al. 2015; Shi et al. 2016; Deng et al. 2018) or substrates—such as humic acids (Lovley et al. 1996), soluble metal ions (Lovley et al. 1991), dimethyl sulfoxide (Gralnick et al. 2006), as well as poised electrodes (Bond et al. 2002).

Transitions in molecular oxygen (O_2_) concentrations over Earth's history have been a major force shaping modern microbial metabolisms (Raymond 2006). As the availability of O_2_ enabled the replacement of many enzymatic reactions central to anaerobic microbial metabolism with aerobic respiratory chains (David and Alm 2011), many lineages have evolved to be obligate aerobes. For example, γ- and *β*-proteobacterial taxa that oxidize ammonia (NH_3_^+^) to nitrite (NO_2_^−^) via O_2_ reduction are considered to be strict aerobes (Koops et al. 2006). However, aerobic ammonia oxidizers are consistently observed in oxygen-limiting and -depleted environments such as anoxic marine zones (Garcia-Robledo et al. 2017) and sediments (Freitag and Prosser 2003; Mortimer et al. 2004), as well as engineered systems (Yu and Chandran 2010). Although a wide range of oxygen affinities for aerobic ammonia-oxidizing bacteria have been reported (Geets et al. 2006), how they are able to persist and survive during oxygen deficiency remains enigmatic.

Many groups of aerobic microbes have been shown to relax their dependence on oxygen using alternative energy metabolisms (Berney et al. 2014). This includes EET, where electrons from central metabolism can be transferred to extracellular minerals such as iron and manganese oxide (Lovley 2012). For example, methane-oxidizing bacteria sharing many metabolic genes with aerobic ammonia-oxidizing bacteria (Khadka et al. 2018) can perform EET under anaerobic conditions using MHCs (McGlynn et al. 2015). Many of these anaerobic methane-oxidizing (ANME) clades are capable of performing syntropy via MHC-driven direct electron transfer between cells. The deeply rooted phylogeny of archaeal ANME clades (Wang et al. 2021, 2022) suggests that MHCs may have a long evolutionary history.

To date, a few studies have reported anaerobic metabolisms in aerobic ammonia-oxidizing bacteria, such as nitrite reduction (Bock et al. 1995; Schmidt et al. 2004; Shaw et al. 2006; Cantera and Stein 2007), but those metabolisms were not linked to any exogenous energy-yielding reaction (Stein 2014). It has also been suggested that *Nitrosomonas* spp. donates electrons to an insoluble electrode in an anoxic electrochemical system, but this, too, has yet to be validated (Vilajeliu-Pons et al. 2018). If aerobic ammonia-oxidizing bacteria can donate extracellular electrons to solid electrodes, they have the phenotypic potential to maintain survival via EET metabolic couplings in the absence of O_2_ in natural settings. We, therefore, hypothesize that ammonia-oxidizing bacteria can switch from aerobic metabolism to anaerobic EET metabolism under anoxic conditions. We further hypothesize that this ability would have evolved early in the evolutionary history of ammonia-oxidizing bacterial clades, as is inferred for EET-capable ANME archaea when both widespread anoxia and oxidized mineral surfaces were likely prevalent (Saito 2012).

To test these hypotheses, we examined the electrochemical response of a model aerobic ammonia-oxidizing bacteria under anoxic conditions. Cyclic voltammetry (CV) and chronoamperometry (CA) were applied to assess the potential capacity for ammonia oxidizers to donate electrons to an electrode, and cytochrome reactive heme staining and metatranscriptomics were employed to identify the presence and activity of their MHCs. Additionally, we performed molecular clock analysis, integrated with reconciliations of genes associated with EET, to estimate the divergence times of ammonia-oxidizing bacterial groups and EET-performing lineages within these groups. Our results show that 1) *Nitrosomonas* may be capable of transferring electrons to solid surfaces in the absence of oxygen and 2) EET capacity in *β-*proteobacterial ammonia oxidizers is ancient, likely acquired by multiple horizontal gene transfers (HGTs) from γ*-*proteobacteria during the Paleo- and Mesoproterozoic (1,556–2,188 Ma and 1,172–1,936 Ma).

## Results and Discussion

### Multiheme EET Cytochromes Are Present in the *Nitrosomonas* Lineage

**FIG. 1.**
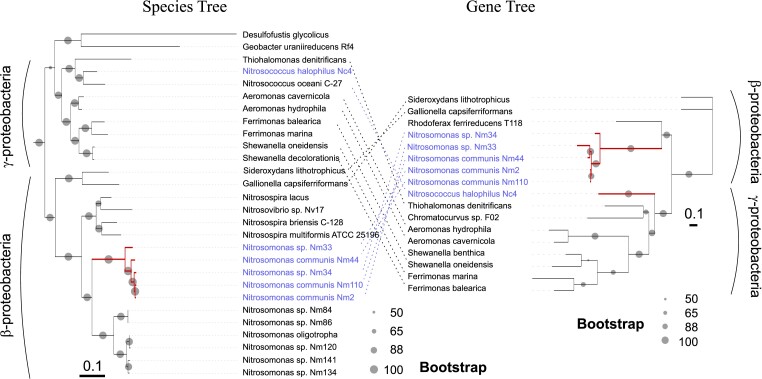
Comparison of phylogenies for species tree and EET metabolism gene tree. Species trees were generated from concatenated alignments of ribosomal protein sequences. EET gene trees were constructed from the concatenated alignments of proteins orthologous to identified EET components in ammonia-oxidizing bacteria, complete MTR pathways, including periplasmic C-type cytochromes (Q8EG35), integral outer-membrane β-barrel proteins (Q8EG34), and outer-membrane-anchored C-type cytochromes (Q8EG33). Ammonia oxidizers with a known complete EET pathway and their phylogenetic distance were colored blue and red, respectively.

EET often relies on a connection between redox and structural proteins (Kumar et al. 2017). For example, a network of proteins physically associated with periplasmic c-type cytochromes, integral outer-membrane β-barrel proteins, and outer-membrane–anchored c-type cytochromes was shown to facilitate EET in *Shewanella oneidensis, Geobacter sulfurreducens, and Rhodopseudomonas palustris* (Shi et al. 2016). We searched the NCBI genome database for genes in ammonia-oxidizing bacteria found in model EET pathways to evaluate their potential EET capability (supplementary table S1, Supplementary Material online). Putative homologs to these known examples were queried using the HMMER search tool NCBI and Ensembl Reference Sequence databases (Gruen et al. 2019). Detected homologs in ammonia-oxidizing bacteria were more similar in sequence to the enzymatic EET machinery of *S. oneidensis* (Coursolle and Gralnick 2010) than *Geobacter* and *Rhodopseudomonas* and were primarily identified in the *Nitrosomonas* genus (supplementary fig. S1, Supplementary Material online). *Nitrosomonas* species possessing the complete EET machinery formed a single clade in the species tree (fig. 1), suggesting that the putative EET metabolism of *Nitrosomonas* appears to be a shared ancestral trait of this group (Coleman et al. 2021). The γ-proteobacterial ammonia oxidizer *Nitrosococcus halophilus* was also identified as carrying a full set of EET metabolism genes. No such homology was detected in the genomes of archaeal ammonia oxidizers.

We then examined MHC containing at least two “C-X-X-C-H” heme-binding motifs in ammonia-oxidizing bacteria and compared the total number of MHC and heme-binding motifs (supplementary fig. S2, Supplementary Material online), as such components constitute the direct electron transport pathway in *S. oneidensis* and *G. sulfurreducens* to reduce extracellular solids (Deng et al. 2018). A total of 25 and 21 MHC carrying 87 and 70 heme-binding motifs were identified in *Nitrosomonas communis* Nm2 and *Nitrosoc occus halophilus* Nc4, respectively, comprising homologs of the EET pathway. Other ammonia oxidizers, on average, contained 16 MHC with 57 heme-binding motifs (supplementary table S2, Supplementary Material online). The high frequency of these homolog motifs suggests that ammonia-oxidizing bacteria *Nitrosomonas* and *Nitrosococcus* can transfer electrons to surfaces in a manner similar to model EET strains. To test this hypothesis, *N. communis* Nm2 and *Nitrosoc. halophilus* Nc4 cultures were grown and examined via bioelectrochemical incubations under oxygen-deficient conditions.

### *Nitrosomonas* May Donate Electrons to Solid Electrodes in an Anaerobic Environment

**FIG. 2.**
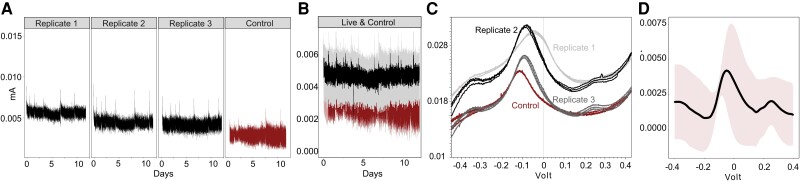
Electrochemical characteristics of *Nitrosomonas communis* on graphite felt electrode. (*A*) Anodic currents were measured using an electrode poised at 0.3 V versus AgCl supplemented with 1 mM NH_4_^+^. (*B*) Mean anodic current from replicate runs of *N. communis* (black) with standard deviation (gray) and abiotic control (red). (*C*) Cyclic voltammograms were measured at a scan rate of 1 mV/s 12 days after initiating the incubation. (*D*) Mean cyclic voltammogram from replicates runs of *N. communis* normalized by subtracting the abiotic control CV as a baseline. The smooth pink layer represents the standard deviation.

We measured the electrochemical response of *N. communis* and *Nitrosoc. halophilus* under anaerobic and ammonium-supplemented conditions (fig. 2*A*). The anodic CA current at 0.3 V versus AgCl was stable at 4.7 ± 2 µA over 12 days in electrochemical incubations of *N. communis*, whereas abiotic control consistently yielded 2.8 ± 0.7 µA over 12 days of incubation (fig. 2*B*). *Nitrosococcus halophilus*, however, did not produce currents that were significantly higher than control incubations (supplementary fig. S3, Supplementary Material online), indicating that *Nitrosoc. halophilus* may not be capable of performing EET under the given conditions. The high frequency of MHCs and the presence of a complete model EET pathway were limited only to a single strain, *Nitrosoc. halophilus*, in the *Nitrosococcus* clade (fig. 1; supplementary fig. S2, Supplementary Material online); this patchwork taxonomic distribution may indicate the presence of a non-EET function that does not require the complete protein machinery (Li et al. 2013; Goodhead and Darby 2015). Future experiments considering varying electric potentials, substrate concentrations, or electrode surface types are needed to reveal the physiology of MHCs in *Nitrosoc. halophilus*. Our data suggest that the graphite felt electrode served as an electron acceptor for *N. communis* in the presence of ammonia, generating a constant anodic current at all culture replicates higher than the controls. However, we did not observe an increase in the anodic current of live incubations, which may show that the microbial electroactivity over this time course was insufficient to support cell growth (Turick et al. 2019). It is plausible that *N. communis* has longer doubling times while using an anaerobic energy metabolism that further limited growth, as previously reported for *N. europaea* (6.5 days in oxic vs. 9 days in anoxic conditions; Kozlowski et al. 2014). The change in cell numbers after 12 days of incubation would likely have been insignificant, given the high initial cell numbers, in line with the results of recent EET incubations with aerobic *Desulfovibrio ferrophilus* (Deng et al. 2018) and our CA profiles. The stable current may also be related to a limited capacity for EET by *N. communis* (Kato et al. 2012) under these experimental conditions. In summary, the observed sustained current in *N. communis* relative to the control, and the lack of elevated current in *Nitrosoc. halophilus* relative to the control supports the hypothesis that *N. communis* might be capable of engaging in EET under anoxic conditions, but further work across a variety of conditions is needed to determine whether *N. communis* can thrive via EET.

To determine the redox-active compounds of *N. communis,* cultures were further analyzed by CV at 1 mV/s scanning rate (fig. 2*C*). Anodic CV peaks that appeared at the abiotic control incubations were consistently lower than at the live incubations, possibly enabling electron transfer to the anode by *N. communis* (Richter et al. 2009). Interestingly, an anodic CV peak was observed in live incubations at the potential of 0.25 V, close to the applied potential in the electrochemical experiments (fig. 2*D*). As all other anodic redox peaks were observed in live and control incubations together, the redox signal at 0.25 V appeared to be affiliated with *N. communis* (Chen et al. 2019). Such redox response hints at a mechanism that might be responsible for the electrochemical activity of *N. communis* involved redox compounds or proteins that developed under O_2_-limited conditions.

### Outer Membrane and Multiheme Cytochromes Were Active in Anode Respiring *N. communis*

**FIG. 3.**
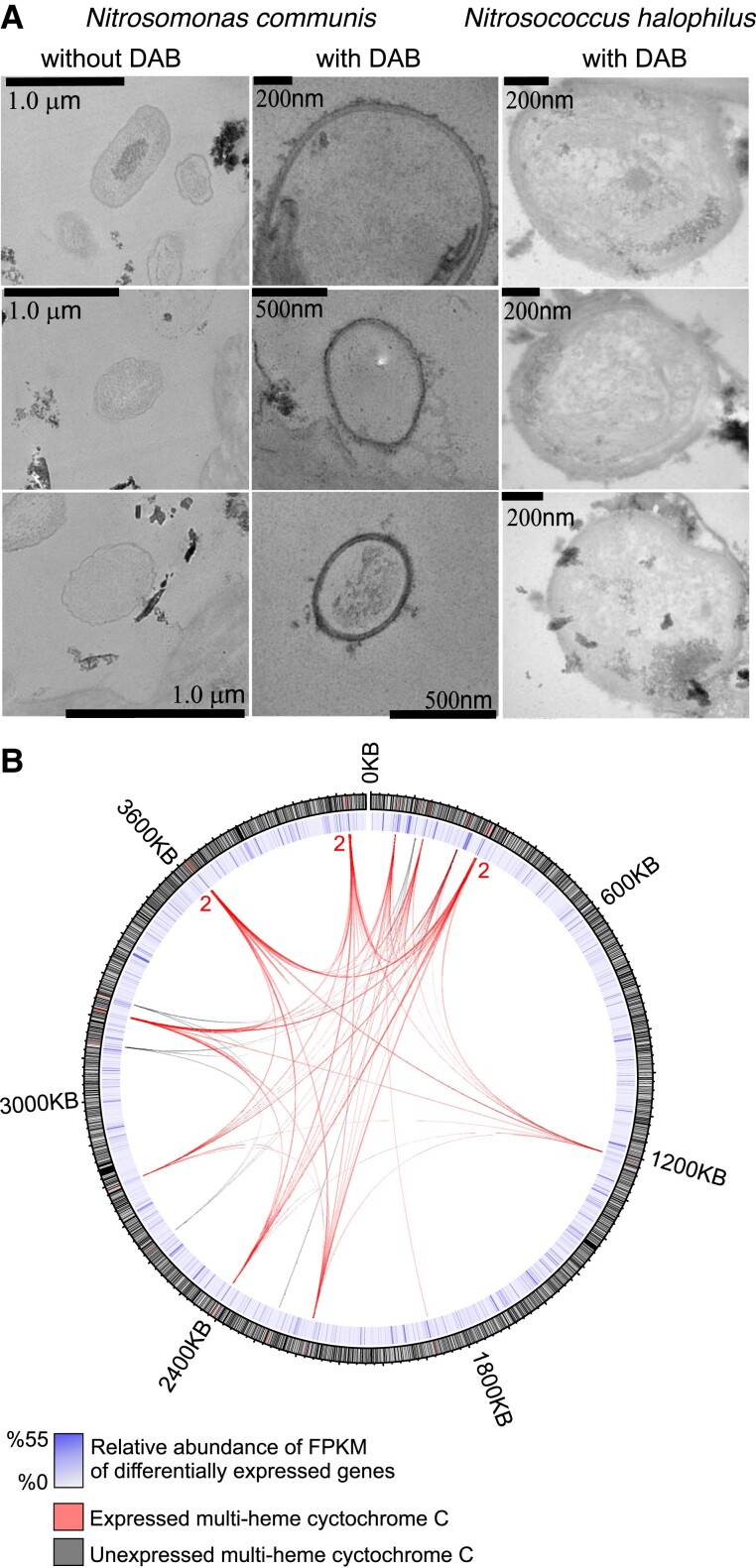
(*A*) Transmission electron microscopy images of *N. communis* and *Nitrosococcus . halophilus* cells stained with cytochrome-reactive DAB-H_2_O_2_ and without DAB. (*B*) Expressed genes of *N. communis* and their expression levels (the inner blue circle) are shown with their genome position (the outer black circle). MHCs having more than one heme-motif are shown as a net inside the circle. Expressed and unexpressed MHCs are colored as red and black, respectively. Red numbers within the circle represents the number of sequential MHC genes in the given genome position.”

Heme staining coupled with TEM imaging was applied to ascertain the presence and distribution of outer membrane heme-containing metalloprotein(s) cytochromes of electro-active *N. communis* after 12 days of incubation without O_2_ (Marshall et al. 2006; Deng et al. 2018). In cytochrome-specific 3,3′-diaminobenzidine (DAB)–H_2_O_2_ staining, heme metal centers catalyze the formation of a DAB polymer that has a high binding affinity to OsO_4_ (Marshall et al. 2006). Thin sections of *N. communis* showed the apparent formation of heme-bound peroxidase, whereas *Nitrosoc. halophilus*, which showed no electrochemical response, exhibited no heme-bound peroxidase activity (fig. 3*A*). This suggests that outer membrane cytochromes of *N. communis* were produced during the electrochemical incubation and potentially involved in the EET mechanism (Hartshorne et al. 2009). The control sections of *N. communis* without the amendment of DAB stain yielded no heme-bound peroxidase signal, indicating that DAB successfully reacted with heme centers of *N. communis* in noncontrol TEM sections. Outer membrane cytochromes are often an essential component of direct EET between microbes and solid surfaces (Kumar et al. 2017); therefore, the detected presence of inducible outer membrane cytochromes further suggests that *N. communis* transferred its electrons to the solid electrode through direct electron transfer. The cytochrome activity of electrochemically incubated *N. communis* was further assessed via metatranscriptomics. Among 25 detected multiheme cytochromes (≥2 heme motifs) in the genome of *N. communis* (supplementary table S3, Supplementary Material online), 19 were expressed after 12 days of electrochemical incubation without oxygen, in line with electrochemical measurements and heme stain assays (fig. 3*B*). Furthermore, the transcript library of *N. communis* revealed the activity of genes affiliated with anaerobic metabolism; the highest expression levels were detected for P460 (supplementary table S4, Supplementary Material online)—a cytochrome converting NH_2_OH to N_2_O (Caranto et al. 2016)—may indicate its potential role in EET activity. In line with the enzymatic mechanism of cytochrome p460 in *N. europhea* (Caranto et al. 2016), NH_2_OH oxidoreductase activity was also detected, which may suggest p460 dependence on NH_2_OH oxidoreductase activity during EET. Although a future isotopic tracing and a comprehensive transcriptomic effort may ultimately provide a complete understanding of the enzymatic EET pathway of *N. communis*, our results suggest that cytochrome P460 can be the enzymatic bridge between ammonia oxidation and the extracellular electron release of *N. communis*. In addition, the low expression of carbon fixation genes (supplementary table S5, Supplementary Material online) was consistent with the nonincreasing anodic currents from CA results (fig. 2*A* and *B*), pointing out that the cell growth was not supported.

### β-Proteobacterial Ammonia-Oxidizing Bacterial Lineages Are over 1.7 Billion Years Old

Although experimental investigations indicated the active metabolism of EET in *Nitrosomonas* spp., a complementary phylogenomics approach can reconstruct the history of EET metabolism in ammonia-oxidizing bacteria, revealing clues about its ecological and planetary significance. To this end, a relaxed molecular clock approach was used to estimate divergence times for *β*-proteobacterial ammonia-oxidizing bacterial lineages inferred to have a shared history of EET metabolisms. We first applied molecular dating to a species tree (Magnabosco et al. 2018; Wolfe and Fournier 2018; Gruen et al. 2019), including *Nitrosomonas* spp., to estimate the divergence time of ammonia-oxidizing bacterial clades and compare these ages with other known EET metabolism groups, such as *Shewanella*. We also directly estimated divergence times for the MtrA protein family tree (supplementary fig. S4, Supplementary Material online) using the calibrations for *Aeromonas*, *Vibrio,* and clade 6 Cyanobacteria (Moore et al. 2019).

As evolutionary models often substantially impact the age estimates of molecular clocks (Dos Reis et al. 2015), we used uncorrelated and auto-correlated branch-specific rate models, as well as uniform and birth-death (BD) priors on the relative age distributions of divergences to estimate the uncertainty of age estimates (Fournier et al. 2021). Any divergence time estimates deep within the Tree of Life will be limited by the paucity of deep, informative fossil calibrations, and the inherent uncertainty of evolutionary rate models. Nevertheless, the obtained posterior age estimates can still be broadly informative and potentially discriminate between hypotheses, such as if a lineage has diverged before or after the great oxygenation event (GOE). Refinements of all age estimates are expected with subsequent improvements in rate models, taxonomic sampling, and the availability of reliable fossil calibrations. A model assessment method was applied to determine the most compatible model with detected HGT events (supplementary fig. S5, Supplementary Material online) as described previously (Fournier et al. 2021). The Cox–Ingersoll–Ross (CIR) process model with a uniform (UNI) prior resulted in the highest compatibility, where 94% of the posterior chronograms tested under CIR + UNI fulfilled HGT constraints (supplementary table S5, Supplementary Material online). Similarly, the CIR model with BD prior showed 93% compatibility, which is very close to the CIR + UNI; we, therefore, considered estimations that covered the time range of both models in our interpretations. The compatibility of other models ranged from 80% to 23%.

**FIG. 4.**
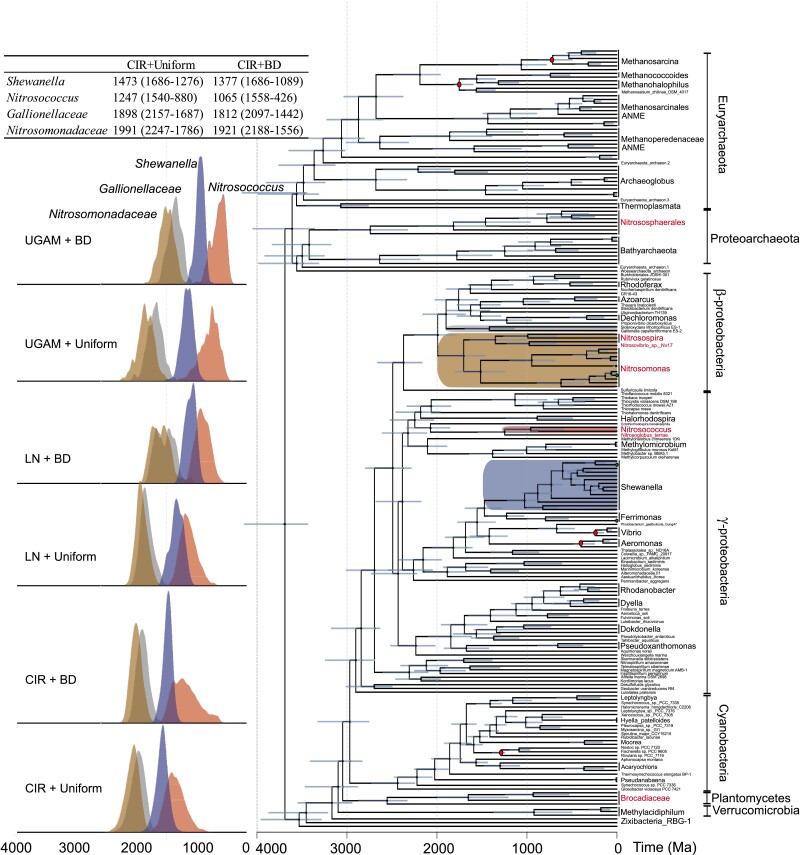
Time-calibrated species tree with ammonia-oxidizing clades highlighted. The depicted chronogram is for the CIR rate model with a uniform distribution, which was selected using the horizontal gene transfer compatibility method (Fournier et al. 2021). Key bacterial clades with putative EET metabolisms are colored from the species tips to their last common ancestral node: Brown-Nitrosomonadaceae, Blue-*Shewanella*, Gray-Gallionellaceae, and Red-*Nitrosococcus*. The red-colored taxa names represent the recognized ammonia-oxidizing clades in this study. Nodes used for calibrations are indicated by red-filled circles. Posterior distributions were generated by sampling the Markov chain Monte Carlo analysis every 1,000 generations, with a 25% burn-in. Blue bars show uncertainty (95% CI). The age distributions of key nodes for the timing of the diversification of Nitrosomonadaceae, Gallionellaceae, *Shewanella*, and *Nitrosococcus* lineages are provided on the left side graph under six different evolutionary models. The mean age estimates for a selection of key nodes under CIR + uniform and CIR + BD models are provided in the table with 95% CI levels.

Molecular clock estimates under the CIR + UNI and CIR + BD models recover *Nitrosomonas* and *Nitrosospira* clades diverging from other *β*-proteobacterial lineages between 1,518 and 1,400 Ma and between 1,355 and 1,246 Ma, respectively (fig. 4). These divergence times represent a younger bound on the acquisition of EET metabolisms by these groups; older bounds are provided by the inferred ages of the *Nitrosomonas* and *Nitrosospira* common ancestors, which we recover as diversifying during the Paleoproterozoic between 2,247 and 1,556 Ma. The origin of *β*-proteobacterial ammonia oxidizers during this interval is consistent with the appearance of their EET metabolism between 2,246 and1,042 Ma, as indicated by the molecular dating analysis of the MtrA gene tree (supplementary fig. S4, Supplementary Material online). The *Shewanella* lineage in possession of EET metabolism was observed to diverge during the Mesoproterozoic between 1,686 and 1,089 Ma. *β*-Proteobacterial ammonia-oxidizing bacteria are inferred to have evolved after the GOE, when the atmospheric oxygen level was more than >5% present atmospheric level (PAL) (Holland 2006; Planavsky et al. 2014; Reinhard and Planavsky 2022) consistent with the proposed shared aerobic ancestry for these lineages. These age estimates suggest that bacterial ammonia oxidizers acquired their metabolisms and diversified at different times during the Proterozoic, whereas the archaeal ammonia oxidizer lineage within Thaumarchaeota (including *Nitrosocaldus*, *Nitrososphaera*, and *Nitrosocosmicus*) had already likely diversified in the Archean, between 2,328 and 3,100 Ma. However, this age estimate is less informed by included secondary calibrations, which are distant from these groups in the species tree and are, therefore, more speculative.

### EET Metabolisms Have Been Distributed across the Tree of Life via Horizontal Gene Transfer

**FIG. 5.**
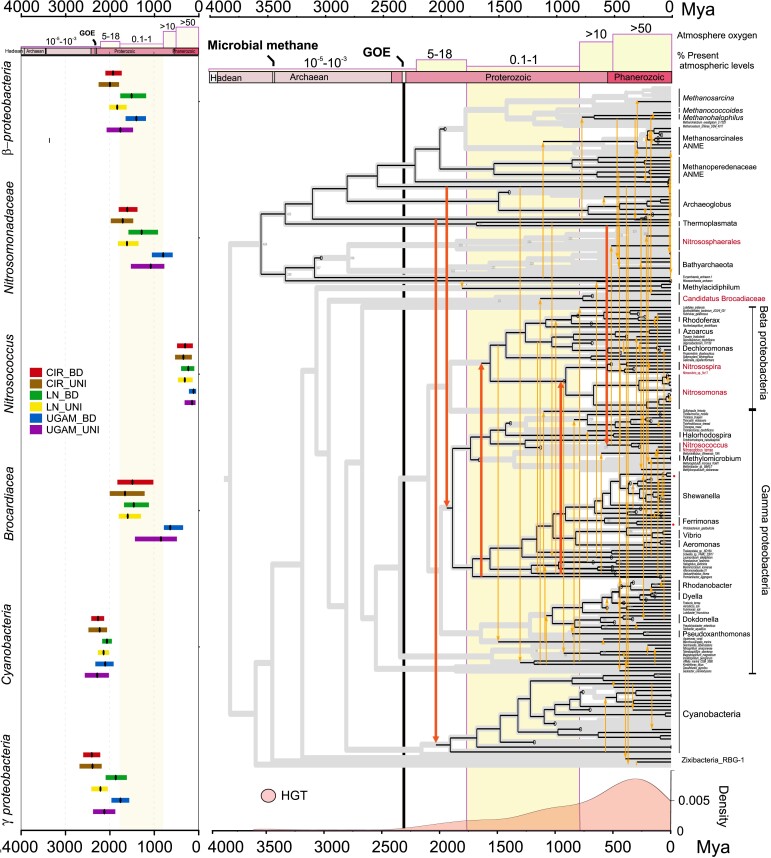
Visualized reconciliation of the electron transfer gene tree (MtrA) onto the dated species tree in this figure constructed from concatenated ribosomal genes. Black lines represent vertical inheritance within the genomes of the species tree, whereas orange lines represent the gene transfer events. Red lines represent the important gene transfer events for bacterial clades and ammonia-oxidizing bacteria (red species-tree tips). Yellow boxes represent atmospheric oxygen levels over time. The yellow box representing the 0.1–1.0% PAL levels within the Proterozoic period is displayed along the reconciliation tree and date summary table to better highlight the evolutionary events during that period. The bottom graph shows the frequency of gene transfer events of the electron transfer gene over time. Taxa are abbreviated as per this figure. A summary of the dates of the reconciled key nodes received the MtrA gene estimated with six evolutionary models is provided on the left.

The Paleoproterozoic appearance of *β*-proteobacterial ammonia-oxidizing bacteria raises the possibility that the EET metabolism detected in extant forms has been conserved for a long period of planetary history. This history can be directly traced through the phylogeny of protein families that are diagnostic for EET metabolic function, and associated molecular clock divergence time estimates can further inform the timing of the acquisition of this function by different groups, albeit with less precision than a species-tree molecular clock. We first compared the protein family tree of periplasmic c-type MHC (MtrA; supplementary fig. S5, Supplementary Material online) to a species tree inclusive of represented taxa using reconciliation implemented in ecceTERA v1.2.4, a technique that infers well-supported speciation, duplication, transfer, and loss events (Libeskind-Hadas et al. 2014; Stolzer et al. 2015) during the evolution of the EET MHC (35; fig. 5). Among all the domain family trees of EET metabolism components, periplasmic MHCs were conserved and widely distributed among the tree of life (supplementary figs. S1, S6, and S7, Supplementary Material online) and hence used to trace the evolution of EET metabolism. Date estimates of key HGT recipient nodes (supplementary fig. S8, Supplementary Material online) were inferred under CIR, LN, and UGAM models with UNI and BD priors (fig. 5).

The reconciled roots for periplasmic MHC, MtrA, map to node 424, the last common ancestor of Euryarchaeota and TACK in figure 5. We further evaluated the EET root position by forcing a posterior root age for the last common ancestor of the MtrA (node 424) in the MtrA gene tree using the age estimates for the crown groups from the species tree as secondary calibrations (supplementary fig. S9, Supplementary Material online). The molecular clock applied to the MtrA gene tree yielded the posterior age estimate of 3,348–4,010 Ma, which is consistent with the age estimate of the reconciled species tree (3,363–4,045 Ma). These broad posterior age distributions are expected, given the limited information available for constraining such deep divergences.

These inferences of deep-rooted ancestry of MtrA from phylogenetic reconciliation are sensitive to both the current sampled diversity of extant sequences and the expected underlying frequencies of gene loss events, as high rates of loss are more compatible with deeper rootings within the species tree. The HGT events and recipients inferred by the reconciliations, on the other hand, are less prone to biases from rooting, reconciliation, and sampling. The reconciliation of the MtrA phylogeny to the species tree suggests that the distribution of MtrA genes in the bacterial tree of life can be explained by major gene transfer events between archaea and bacteria (fig. 5, red arrows). The earliest HGTs are mapped from 1) Thermoplasmata to Cyanobacteria and 2) Methanoperedenaceae to γ-proteobacteria. Each of these HGT events is inferred to have occurred after the GOE (2,175 ± 90 Ma to Cyanobacteria and 2,120 ± 266 Ma to γ-proteobacteria) (fig. 5). Reconciliation analysis suggests that *β*-proteobacteria acquired their periplasmic EET MHC from γ-proteobacteria in multiple HGT events, including one HGT to the common ancestor of *Nitrosomonas* and *Nitrosospira* (fig. 5). This HGT to Nitrosomonadaceae consistently occurs during the time of presumed oxygen limitation (Holland 2006; Planavsky et al. 2014) in all model estimations (fig. 5). The ancestral *β*-proteobacterial ammonia oxidizer, therefore, initially acquired the anaerobic EET metabolism early in Earth's history of atmospheric oxygenation, when transient oxygen limitation was likely commonplace. As in Methanoperedenaceae ANME, our study indicates that EET metabolism appears to be still functional in *β*-proteobacterial ammonia-oxidizers today.

Integrating the results of these experimental and phylogenomic studies with the geochemical history of oxygen suggests a period of ecological and metabolic transition during the Proterozoic, somewhat ironically marked by an expansion of anaerobic oxidation metabolisms. Our experimental findings show that a modern aerobic bacterial ammonia oxidizer may survive oxygen-limiting conditions through EET, which enables the oxidation of inorganic solid-phase electron acceptors. Future studies should aim to establish the growth rates of *N. communis* in anoxic conditions and elaborate on the range of mineral oxides capable of supporting growth. In addition, our analyses reveal that multiple HGT events from γ-proteobacteria to *β*-proteobacteria and Nitrosomonadaceae are inferred to have occurred during the Proterozoic, providing ammonia oxidizing lineages with archaeal EET metabolisms. The timing of these evolutionary innovations may be due to a regime of fluctuating anoxic–oxic interfaces that persisted until the late Proterozoic (742–542 Ma) (Saito 2012) and thus might explain why aerobic ammonia oxidizers are consistently observed in oxygen-limiting and -depleted environments (Freitag and Prosser 2003; Mortimer et al. 2004; Yu and Chandran 2010; Garcia-Robledo et al. 2017). This ordering of evolutionary events, specifically, HGT of EET encoding genes within groups of aerobic bacterial ammonia oxidizers, hints that electrogenic ammonia oxidation is not ancestral to aerobic ammonia oxidation. This is in line with the vast increase of oxidized minerals after the GOE, which could serve as electron acceptors for EET (Saito 2012). Our homology searches did not identify EET signals in archaeal ammonia oxidizers. One possibility is that these groups have lost their ancestral MHCs following the gain of other metabolic adaptations (Kraft et al. 2022) to cope with O_2_ fluctuations and limitations, as ammonia-oxidizing archaea have been consistently detected in marine oxygen-minimum zones and oxygen-deficient sediments (Molina et al. 2010; Stahl and de la Torre 2012; Sollai et al. 2019). Additional anaerobic pathways may remain to be investigated among other groups of aerobic microbes, which could potentially be revealed using similar electrode-based culture enrichment methods and phylogenomics.

## Materials and Methods

### Bacterial Strains and Culture Conditions

The strains used for this study were *N. communis Nm2* (acquired from Dr. Lisa Stein's lab) and *Nitrosoc. halophilus* (Japan Collection of Microorganisms [JCM], 30413). *Nitrosomonas communis* was grown in oxic media containing (g per liter): (NH_4_)_2_SO_4_: 0.66; NaCl: 0.58; KH_2_PO_4_: 0.05; MgSO_4_.7H_2_O: 0.05; CaCl_2_.2H_2_O: 0.14; and KCl: 0.07. The pH was adjusted to 7.6. *Nitrosococcus halophilus* was cultivated with JCM 1056 media. Cultures were incubated at 28 using a rotary shaker.

### Electrochemical Incubation and Analysis

Two-chamber electrochemical reactors were constructed using 100 ml glass Schott bottle sealed with butyl stopper. The compartments were separated using Nafion tubing (PermaPure LLC, USA), and the anode was equipped with a custom-made AgCl reference electrode (KCl 3.5 M), and carbon felt (3 cm length, 3 cm width, 1.12 cm thickness, 0.72 m^2^/g surface area, Alfa-Aesar, USA) working electrode attached to a titanium wire (0.06 cm diameter; Sigma), whereas a platinum wire was used as the counter electrode at the cathode. Continuous gassing of N_2_/CO_2_ was provided to ensure the constant mixing, anaerobiosis, and pH, in reactors. Culture media were used as the electrolyte after replacing (NH_4_)_2_SO_4_ to NH_4_Cl and MgSO_4_.7H_2_O to MgCI_2_ and degassing with N_2_/CO_2_ for a day. *Nitrosomonas communis* and *Nitrosoc. halophilus* were filtered (0.22 µM) from the aerobic media, washed, and resuspended in 5 ml anoxic electrolyte in an anaerobic chamber. Electrolyte solution then gently vortexed with carbon felt working electrode and left for 15 min of settling. The electrochemical reactor was then sealed with the culture-attached working electrode, remaining electrolyte, and all other components inside the anaerobic chamber and placed for the CA run while purging N_2_/CO_2_.

For CA measurements, VMP3 multichannel potentiostat (BioLogic Company, France) was used on electrochemical reactors with an applied anode potential of 0.3 V (vs. AgCl). Along with triplicate reactors of *N. communis* and *Nitrosoc. halophilus*, parallel control reactors were operated without biomass. Identical voltage settings were used for both inoculated and control reactors. At the end of the 12 days CA run, CV was applied to characterize the redox behavior of the cultures. The scan range of CV was from 0.4 V to −0.4 V, with a scan rate of 1 mV/s, using the VMP3 multichannel potentiostat. A low scan rate 1 mV/s was applied to minimize the background capacitive current and the kinetic limitations of interfacial electron transfer between the microbial cells and the electrode (Marsili et al. 2008). The mean CV of *N. communis* was calculated with QSoas (https://bip.cnrs.fr/groups/bip06/software/) after subtracting the abiotic CV from all biotic CVs as a baseline.

### Cytochrome Reactive Staining and Transmission Electron Microscopy

*Nitrosomonas communis* and *Nitrosoc. halophilus* cells were fixed from 1 × 1 × 1 cm^3^ piece of working electrode at the end of 12 days of incubation using deaerated solutions containing 2% paraformaldehyde and 2.5% glutaraldehyde on ice. After fixation, cells were dislodged from the electrode material by gentle vortexing, and suspended cells were filtered through 0.22 µM Nuclepore Track-Etched polycarbonate filters. Filters were washed 5 × with 1.5 ml 50 mM Na^+^-Hepes (pH 7.4, 35 g/liter NaCl). DAB-H_2_O_2_ and OsO_4_ staining were applied as described previously (McGlynn et al. 2015). After dehydration through a graded series of ethanol, DAB and OsO4 stained filters were placed first into 1:1 propylene oxide/ethanol solution and then into 100% propylene oxide solution. Epon812 was used as an embedding resin for electron microscopy and polymerized at 60 °C overnight with the filters after preincubation using 100 µl ethanol/Epon 812 (1:1) overnight.

### Transcriptomic Library and Analysis

Total RNA extraction was applied to 2 × 2 × 2 cm^3^ working electrode at the end of the 12 days incubation. The electrode material was immediately embedded into a “Trizol Reagent” (ThermoFisher Scientific) in FastPrep Tubes with Zirconia/Silica beads. Bead beating with MP Biomedicals FastPrep −24 for 90 s at 10 m/s was then applied, followed by 200 µl chloroform addition to promote phase separation. With the clear phase separation and formation using centrifugation at 12,000 × for 15 min at 4 °C, the total RNA was extracted from the clear aqueous phase of the solution using Direct-zol RNA MiniPrep kit (Zymo Research) according to the manufacturer's instructions. rRNA was removed with Ribo-Zero Plus kit (Illumina Inc.), and the cDNA sequencing library was prepared using the TruSeq Stranded mRNA Library Prep Kit (Illumina), following the manufacturer's guidelines. Sequencing was applied with NovaSeq 6000, S2 100PE generating ∼50 M (25 M per read) million 100 bp pair-end reads per library.

Sequence quality control tool FastaQC (Andrews 2010) and sequence preprocessing tool Trimmomatic (Bolger et al. 2014) were used to perform the quality check and process the raw paired-end reads. The published genome of *N. Communis* Nm2 (GenBank: GCA_001007935.1, RefSeq: GCF_001007935.1) was used as the reference genome and transcripts for this study. The alignments of processed pair-end reads were performed using HISAT2 (Kim et al. 2019), and aligned sam files were then sorted and processed using SAMtools suit. The transcript counts were then performed using Stringtie2 (Kovaka et al. 2019). Circus was used to visualize genome maps, transcripts, and expression levels. MHCs of *N. Communis* Nm2 were detected using the online MOTIF search tool of GenomeNet (https://www.genome.jp/tools/motif/MOTIF2.html).

### Construction of Sequence Data Sets

A network of redox and structural proteins driving EET are well characterized in a few model organisms: the metal-reducing (Mtr) pathway of *S. oneidensis MR-1* (Coursolle and Gralnick 2010), the porin–cytochrome (Omc) pathways of *G. sulfurreducens* (Liu et al. 2015), the phototrophic iron oxidation (Pio) pathway of *R. palustris TIE-1* (Bird et al. 2014), and the metal-oxidizing pathway (Mto) pathway of *Sideroxydans lithotrophicus ES-1* (Liu et al. 2012). The NCBI nonredundant protein database was queried using BLASTp and HMMER v3.3.2 for homologs of the Mtr, Omc, Mto, and Pio proteins in ammonia-oxidizing taxa having a sequenced genome. Homologs of MtrA, MtrB, and MtrC were additionally queried in the NCBI protein nr database in prokaryotes having sequenced genomes. No Mtr proteins were found in archaeal ammonia oxidizers. Sequences of each protein family were aligned using the MAFFT algorithm version 7.313 (https://mafft.cbrc.jp/alignment/software/; Katoh et al. 2005) with parameters –ep 0 –genafpair –maxiterate 1000 and inspected manually for the presence of gaps and misalignment.

### Construction of Species Tree and Gene Trees

Aerobic ammonia-oxidizing bacteria are distributed across the *α*, *γ*, *β*, *δ* Proteobacteria, the Chloroflexi, and the Nitrospirota (Klotz and Stein 2008); among them, the *β* proteobacterial clade, containing the *Nitrosomonas* and *Nitrosospira* genera, have become model for ammonia-oxidizing bacteria, and all isolated strains in this clade affiliate to a monophyletic evolutionary group (Kowalchuk and Stephen 2001). Complete genomes of 21 physiologically proven ammonia-oxidizing bacteria with 11 strains of *Nitrosomonas* clade and 3 strains of *Nitrosospira* clade were collected to form a species database. All ammonia oxidizers with genomes containing detected EET homologs were selected to construct a gene and species tree. We only used complete and nearly complete genomes in public databases, as the initial goal of our study was to focus on the evolutionary history of this pathway, grounded in experimentally determined function. We agree that extending this work to include metagenome-derived sequences containing detected homologs to EET proteins would potentially provide a broader history of these protein families. However, excluding these sequences does not “bias” our results, per se, as none of our conclusions depend upon the proposed rarity or uniqueness of this metabolism within bacteria; in fact, as is the case with all microbial phylogenetics work, the true diversity likely greatly exceeds that within any current database, complete genome, or metagenome. A species tree based on single-copy universally conserved genes was constructed for the 208 microbial species using the “bcgTree” (https://github.com/molbiodiv/bcgTree; Ankenbrand and Keller 2016) pipeline, specifically including groups known to be ammonia oxidizers. Predicted protein-coding sequences of 208 species were queried against a database of hidden Markov model profiles of the single-copy universal genes (Dupont et al. 2012; supplementary table S6, Supplementary Material online) found in over 95% of the proteomes using HMMER v3.3.2. Best matches remaining after a gene-specific cutoff (48; supplementary table S6, Supplementary Material online) were retained and aligned using MUSCLE v3.8.1551 (https://www.drive5.com/muscle/) with default settings. The resulting alignments were filtered with Gblocks v0.91b (https://www.biologiaevolutiva.org/jcastresana/Gblocks.html; Talavera and Castresana 2007) and concatenated with AMAS. (https://github.com/marekborowiec/AMAS; Borowiec 2016). A maximum likelihood phylogenetic tree was constructed from the alignment with RAxML v8.2.12 (https://github.com/stamatak/standard-RAxML; Kozlov et al. 2019). A subtree including all proteobacterial ammonia oxidizers included in the constructed species tree was used in figure 1.

The MtrA proteins retrieved from the proteomes of taxa included in the species tree were aligned using the MAFFT algorithm version 7.313. The MtrA tree was constructed with IQ-Tree version 1.6.6 (Nguyen et al. 2015) with the best fitting substitution model (WAG) chosen according to the bayesian information criterion, with 1,000 ultrafast bootstraps generated to assess bipartition supports.

### Divergence Time Estimation

Molecular clock analysis for the species tree dataset was performed using Phylobayes v4.1c (Lepage et al. 2007) under the CAT20 model, BD and UNI tree priors, and uncorrelated gamma distributed (UGM), auto-correlated lognormal (LN), and autocorrelated CIR process rate models. Molecular clock analysis for the MtrA dataset was performed using Phylobayes v4.1c under an auto-correlated CIR process rate model and UNI tree prior, using WAG as the substitution model (as identified as best-fitting in IQ-Tree). By dating the MtrA gene tree with the secondary calibrations acquired from the dated species tree, we compared and evaluated the age of the reconciled root of the MtrA metabolism with two dating approaches. Both analyses were run using two chains; after chain convergence (effective size > 50, variable discrepancies < 0.30), the initial 20% of sampled generations were discarded as burn-in, and trees and posterior probability support values were calculated from completed chains. The sampled age estimates were then compared with and without posterior likelihood calculations.

### Age Constraints

Secondary calibrations were applied to the divergence times of archaeal and bacterial groups within the species tree and MtrA gene tree based on previously published age estimates. For the species tree, we applied a time constraint for Archaea within 1) the Methanosarcina group and 2) the split of *Methanococcoides* and *Methanohalobium* groups using the posterior divergence time estimates based on the ribosomal alignment partitions reported in Wolfe et al. (Wolfe and Fournier 2018). We also applied time constraints for bacteria for the crown diversifications of 1) the genus *Aeromonas* (Sanglas et al. 2017) and 2) the genus of *Vibrio* (Lin et al. 2018), and divergence of total-group Nostocales in our tree defined as the clade including *Nostoc*, *Fischerella*, and *Rivularia* (Fournier et al. 2021). The root was calibrated with a prior of 3.9 ± 0.23 Ga ranging from 4.36 to 3.44 Ga as calculated previously (Horodyski and Allan Donaldson 1980; Wolfe and Fournier 2018). All calibrations are listed in supplementary table S7, Supplementary Material online. The MtrA tree was similarly calibrated using the estimated ages of nine crown groups congruent in the reconciled species–gene tree (supplementary table S8, Supplementary Material online).

### Phylogenetic Reconciliation

Reconciliation of the MtrA gene tree to the species tree was performed with ecceTERA v1.24, which employs species-tree-aware gene trees via the joint amalgamation method (Jacox et al. 2016). The ecceTERA algorithm follows Duplication-Transfer-Loss (DTL) model and considers the evolutionary events of speciation, gene duplication, speciation-loss, HGT, and transfer-loss between chosen strains and from/to unsampled/extinct species. In the DTL model, reconciliation is performed using a maximum parsimony formulation aiming to find a reconciliation that minimizes the total cost of the incurred events. Time estimates for the main events in mtrA evolution were generated by mapping the most parsimonious reconciliations of the gene trees against the time-calibrated species tree. The phylogenetic reconciliation was performed using the following sets of DTL (δ, τ, λ) event cost vectors: (1, 1, 1) = A, (1, 3, 1) = B, without (A00, B00), and with (A10, B10) transfers to/from a dead lineage or the Pareto-optimal strategies 1 = C01 and 3 = C03, as previously published (Libeskind-Hadas et al. 2014; Jacox et al. 2016; Waglechner et al. 2019). A reconciliation viewer, SylvX (http://www.sylvx.org/), was used to interpret and compare reconciliations.

### Application of Horizontal Gene Transfer-Based Evolutionary Model Assessment

HGT events to key nodes were selected using the MtrA gene and species tree reconciliations (supplementary fig. S6, Supplementary Material online). For each HGT selected, “compatible” chronograms sampled after burn-in showing the donor being older than the recipient, were selected. The percentage of compatible chronograms was then calculated for each HGT (supplementary table S5, Supplementary Material online) as described by Fournier et al. (Fournier et al. 2021). Posterior age distributions were then generated from selected compatible chronograms.

## Supplementary Material

msad161_Supplementary_Data

## Data Availability

Supplementary data, Supplementary Material online files are available at https://github.com/ardagulay/. All sequence data have been deposited at NCBI GenBank under Biosample accession number SAMN31896628.
