# Supplementary material for: Proterozoic Acquisition of Archaeal Genes for Extracellular Electron Transfer: A Metabolic Adaptation of Aerobic Ammonia-Oxidizing Bacteria to Oxygen Limitation
Source: Mol Biol Evol. 2023 Jul 13;40(8):msad161. doi: 10.1093/molbev/msad161 (PMC10415592; doi:10.1093/molbev/msad161)
Supplement: msad161_Supplementary_Data [file msad161_supplementary_data.docx]

**SUPPLEMENTARY MATERIAL**

**Proterozoic Acquisition Of Archaeal Genes For Extracellular Electron Transfer:**

**A Metabolic Adaptation Of Ammonia-Oxidizing Bacteria To Oxygen Deficiency**

Arda Gülay^1,2^, Greg Fournier^3^, Barth F. Smets^2^, Peter R. Girguis^1^

^1^ Department of Organismic and Evolutionary Biology, Harvard University, Cambridge, MA, United States **e-mail:** ardagulay@fas.harvard.edu, pgirguis@oeb.harvard.edu

^2^ Department of Environmental Engineering, Technical University of Denmark, Building 113, Miljøvej, 2800 Kgs Lyngby, Denmark, **e-mail:** argl@env.dtu.dk, bfsm@env.dtu.dk

^3^ Department of Earth, Atmospheric and Planetary Sciences, Massachusetts Institute of Technology, Cambridge, MA, USA, **e-mail:** [g4nier@mit.edu](mailto:g4nier@mit.edu)

**Supplementary Table 1:** Model EET proteins used in this study for homology search

| Protein Uniprot ID | Protein Name | Strain |
| --- | --- | --- |
| Q8E8S0 | CymA | Shewanella oneidensis (strain MR-1) |
| Q8EG33 | MtrC | Shewanella oneidensis (strain MR-1) |
| Q8EG34 | MtrB | Shewanella oneidensis (strain MR-1) |
| Q8EG35 | MtrA | Shewanella oneidensis (strain MR-1) |
| Q749L1 | OmcC | Geobacter sulfurreducens (strain ATCC 51573 / DSM 12127 / PCA) |
| Q748W7 | OmcA | Geobacter sulfurreducens (strain ATCC 51573 / DSM 12127 / PCA) |
| Q749K5 | OmcB | Geobacter sulfurreducens (strain ATCC 51573 / DSM 12127 / PCA) |
| Q74G83 | ppcB | Geobacter sulfurreducens (strain ATCC 51573 / DSM 12127 / PCA) |
| G5EBD6 | ppcA | Geobacter sulfurreducens (strain ATCC 51573 / DSM 12127 / PCA) |
| Q74FJ0 | OmcE | Geobacter sulfurreducens (strain ATCC 51573 / DSM 12127 / PCA) |
| Q74A86 | OmcS | Geobacter sulfurreducens (strain ATCC 51573 / DSM 12127 / PCA) |
| Q74DC2 | OmpB | Geobacter sulfurreducens (strain ATCC 51573 / DSM 12127 / PCA) |
| Q749T5 | OmpC | Geobacter sulfurreducens (strain ATCC 51573 / DSM 12127 / PCA) |
| A1EBT2 | PioA | Rhodopseudomonas palustris (strain TIE-1) |
| A1EBT3 | PioB | Rhodopseudomonas palustris (strain TIE-1) |
| A1EBT4 | PioC | Rhodopseudomonas palustris (strain TIE-1) |


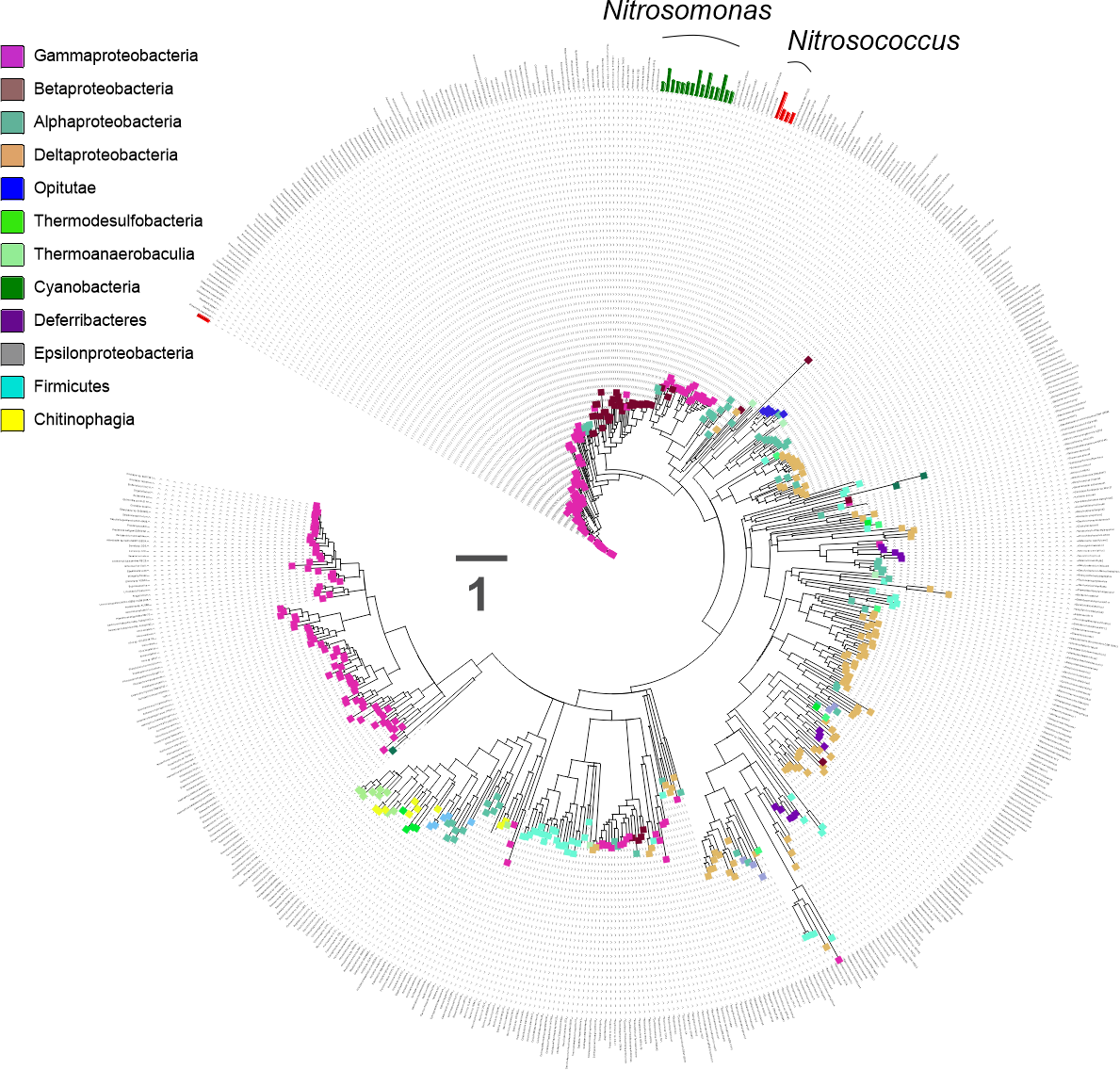


**Supplementary Figure 1**: The phylogenetic tree of homologs of periplasmic c-type cytochromes (mtrA) driving EET in *Shewanella Oneidensis* in the tree of bacterial life.


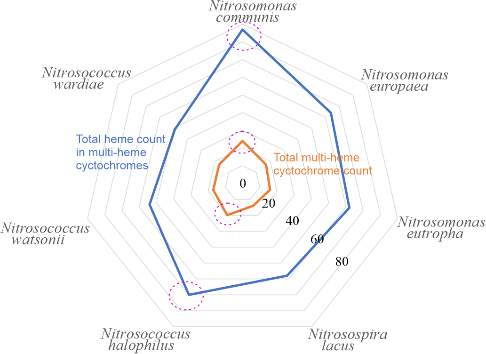


***Supplementary Figure 2*** *Comparison of total multi-heme cytochrome- and total heme-binding motif- counts of selected Nitrosomonas and Nitrosococcus strains*

**Supplementary Table 2:** Heme quantity and number of MHC in selected ammonia-oxidizing bacteria

| Nitrosomonas communis | Nitrosomonas europaea | Nitrosomonas eutropha | Nitrosospira lacus | Nitrosococcus halophilus | Nitrosococcus watsonii | Nitrosococcus wardiae |
| --- | --- | --- | --- | --- | --- | --- |
| 10 | 8 | 8 | 8 | 10 | 8 | 8 |
| 8 | 8 | 8 | 8 | 10 | 8 | 5 |
| 8 | 8 | 8 | 8 | 8 | 5 | 4 |
| 8 | 4 | 4 | 4 | 5 | 4 | 4 |
| 4 | 4 | 4 | 4 | 4 | 4 | 3 |
| 4 | 4 | 4 | 4 | 4 | 3 | 3 |
| 4 | 4 | 4 | 4 | 3 | 2 | 2 |
| 4 | 4 | 4 | 4 | 2 | 2 | 2 |
| 4 | 3 | 3 | 3 | 2 | 2 | 2 |
| 3 | 2 | 2 | 2 | 2 | 2 | 2 |
| 3 | 2 | 2 | 2 | 2 | 2 | 2 |
| 3 | 2 | 2 | 2 | 2 | 2 | 2 |
| 2 | 3 | 2 | 2 | 2 | 2 | 2 |
| 2 | 2 | 2 | 3 | 2 | 2 | 2 |
| 2 | 2 | 3 |  | 2 | 2 | 2 |
| 2 | 2 | 2 |  | 2 | 2 | 2 |
| 2 | 2 |  |  | 2 | 2 | 2 |
| 2 |  |  |  | 2 |  |  |
| 2 |  |  |  | 2 |  |  |
| 2 |  |  |  | 2 |  |  |
| 2 |  |  |  |  |  |  |
| 2 |  |  |  |  |  |  |
| 2 |  |  |  |  |  |  |
| 2 |  |  |  |  |  |  |


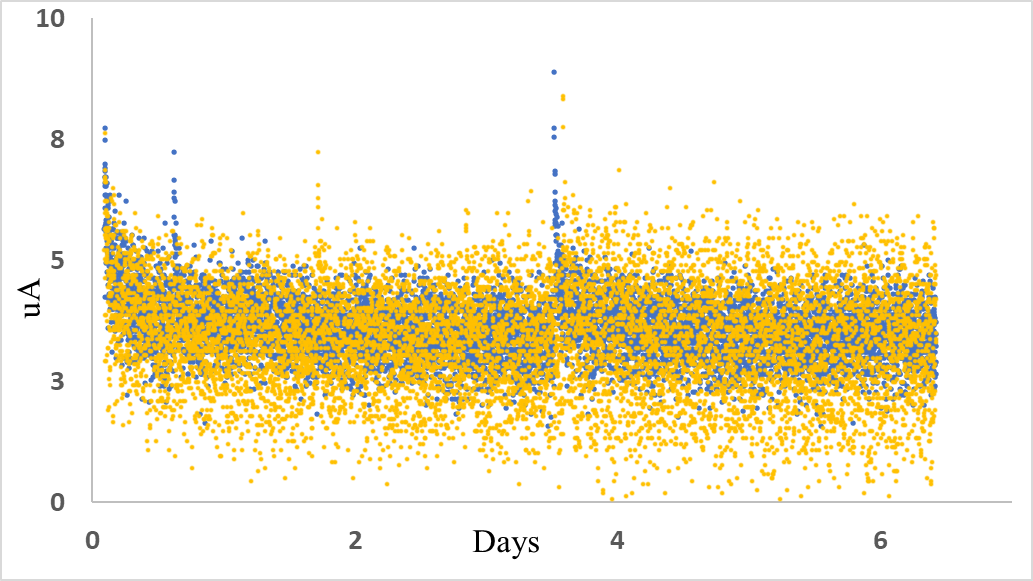


**Supplementary Figure 3**: Electrochemical characteristics of *Nitrosococcus halophilus* on graphite felt electrode. Anodic currents were measured using an electrode poised at 0.3V versus AgCl supplemented with 1 mM NH_4_^+^. Control (yellow color) yielded similar current values as live incubations.

**Supplementary Table 3** Detected multi-heme cytochromes in *Nitrosomonas communis* Nm2

**Supplementary Table 4:**Genes affiliated with anaerobic energy metabolism in *Nitrosomonas communis* Nm2 genome and their expression levels

**Supplementary Table 5:** Genes affiliated with carbon fixation metabolism in *Nitrosomonas communis Nm2* genome

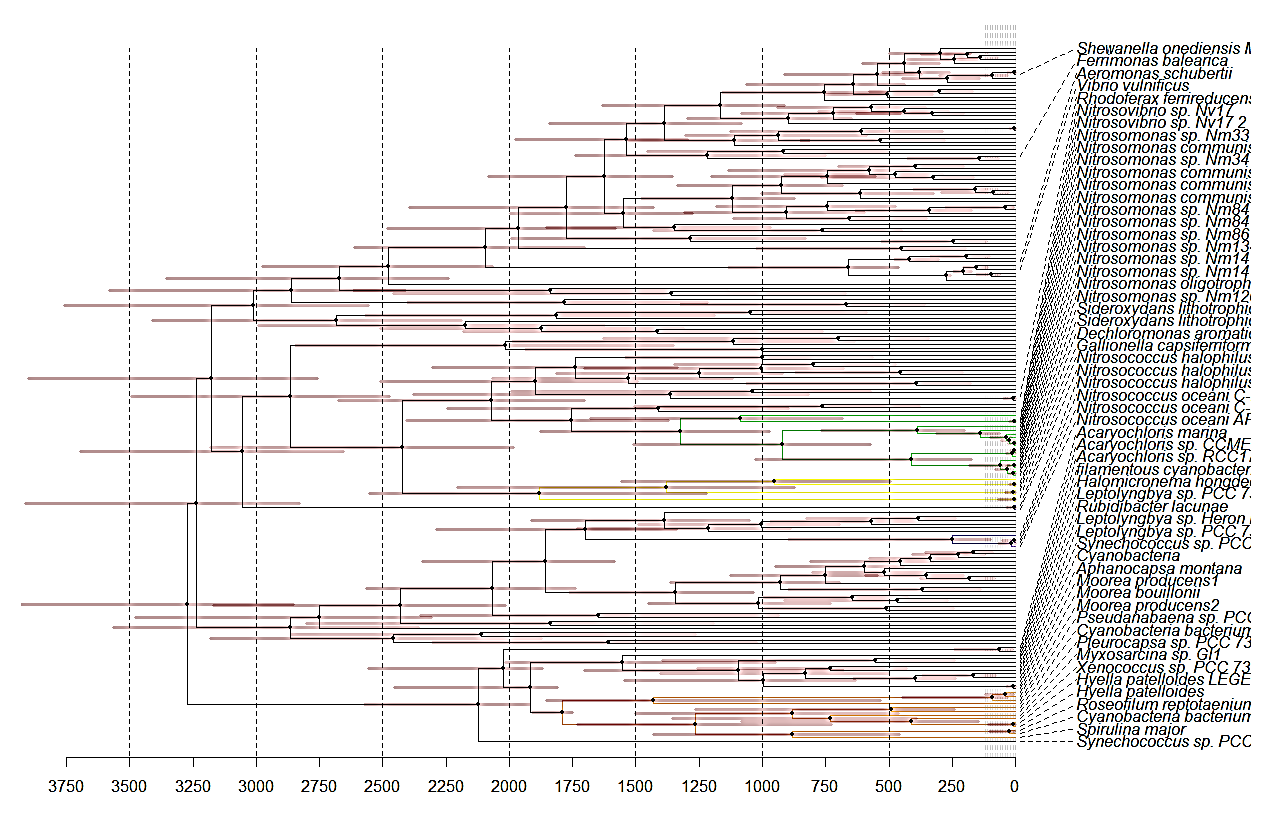


**Supplementary Figure 4** Chronogram depicting the phylogenetic relationships between mtrA homologs, and posterior age estimates obtained under the prior. Ages were estimated in PhyloBayes using the WAG substitution model and the UGM molecular clock model. Horizontal bars on the nodes indicate 95% CIs.


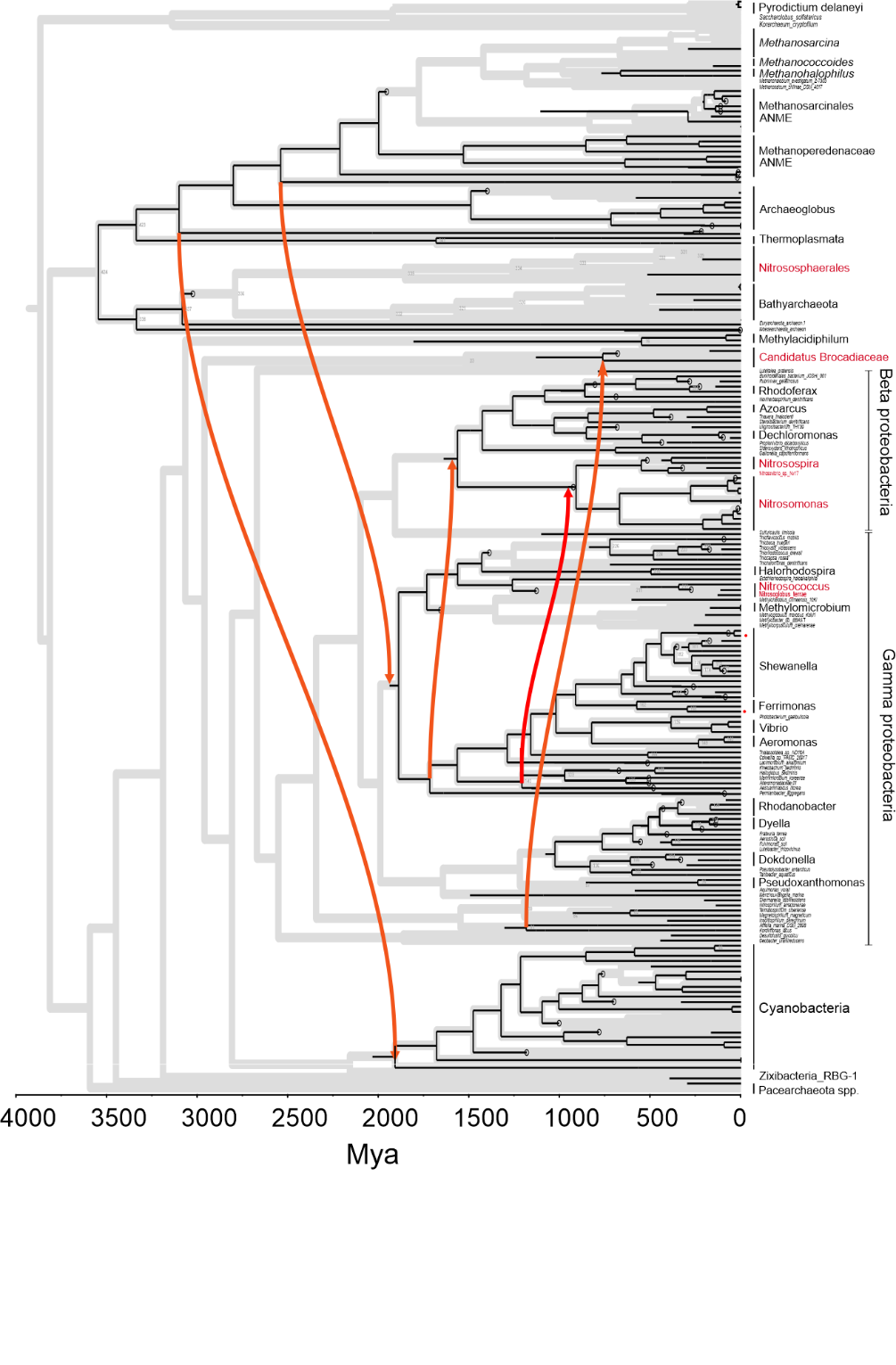


**Supplementary Figure 5** Visualized reconciliation of the electron transfer gene tree (MtrA) onto the dated species tree (grey) constructed from concatenated ribosomal genes. Black lines represent vertical inheritance within the genomes of the species tree, whereas red lines represent the selected gene transfer events for bacterial clades and ammonia-oxidizing bacteria (Red and black bold species-tree tips). HGTs are mapped with arrows from older (donor) node to younger (recipient nodes).

**Supplementary Table 5:** Compatibility results based on the HGT events in Figure 1

|  | Compatibility |
| --- | --- |
| CIR+BD | 0.93 |
| CIR+UNIFORM | 0.94 |
| LN+BD | 0.23 |
| LN+UNIFORM | 0.52 |
| UGAM+BD | 0.80 |
| wUGAM+UNIFORM | 0.72 |


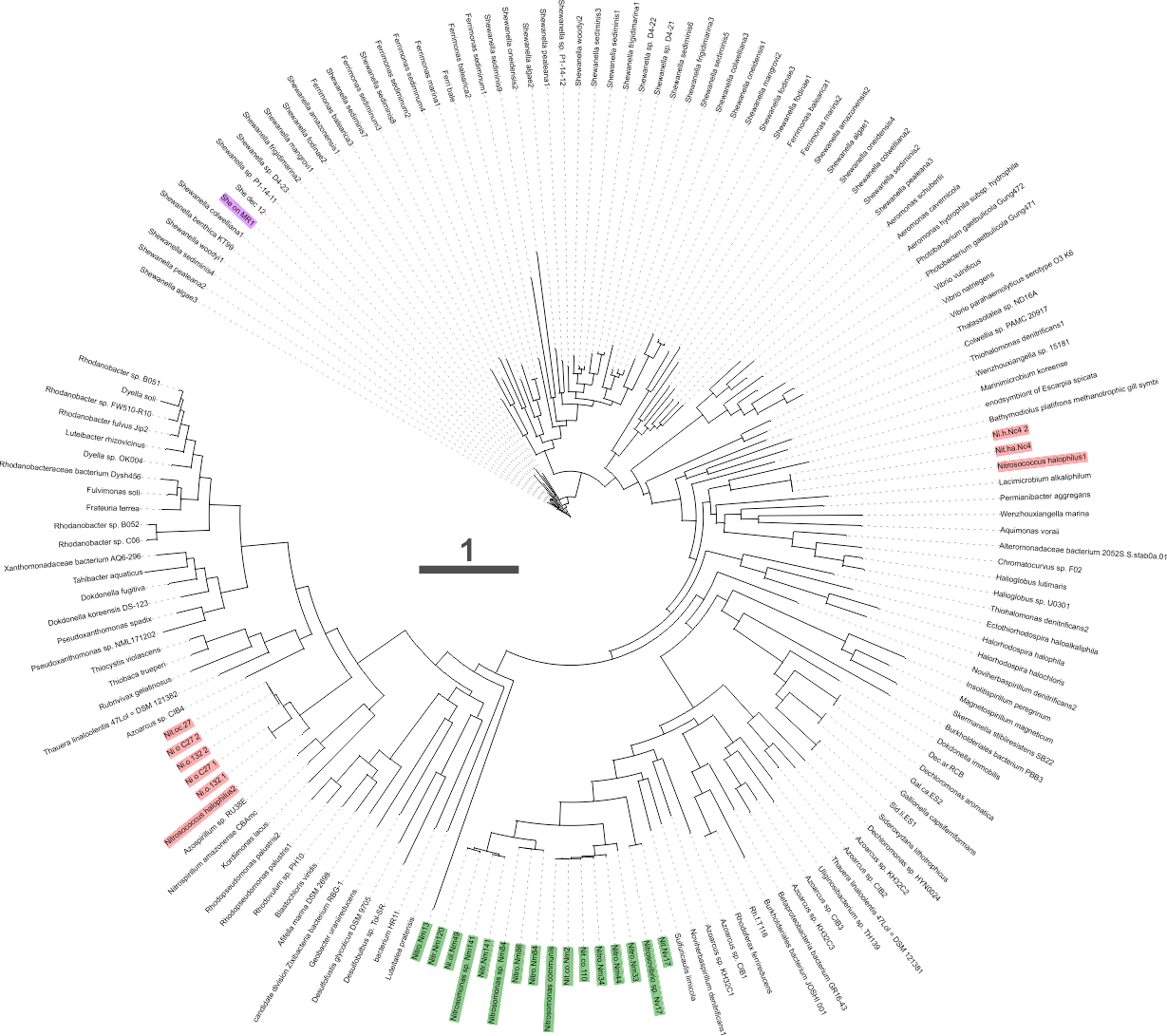


**Supplementary Figure 6:** The phylogenetic tree of homologs of integral outer-membrane β-barrel proteins (mtrB) driving EET in *Shewanella Oneidensis* in the tree of bacterial life.


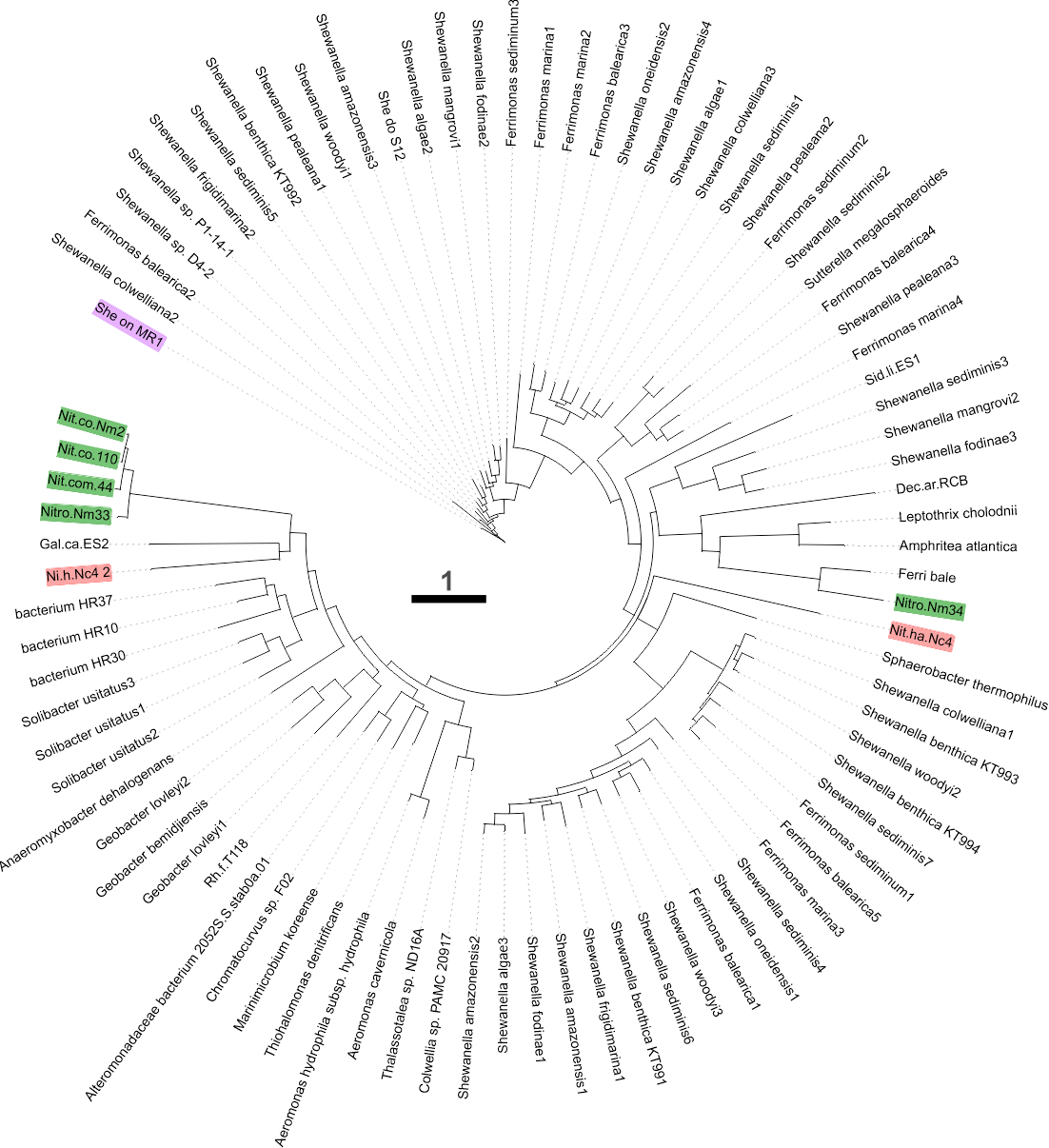


**Supplementary Figure 7:** The phylogenetic tree of homologs of outer-membrane-anchored c-type cytochromes (mtrC) driving EET in *Shewanella Oneidensis* in the tree of bacterial life.


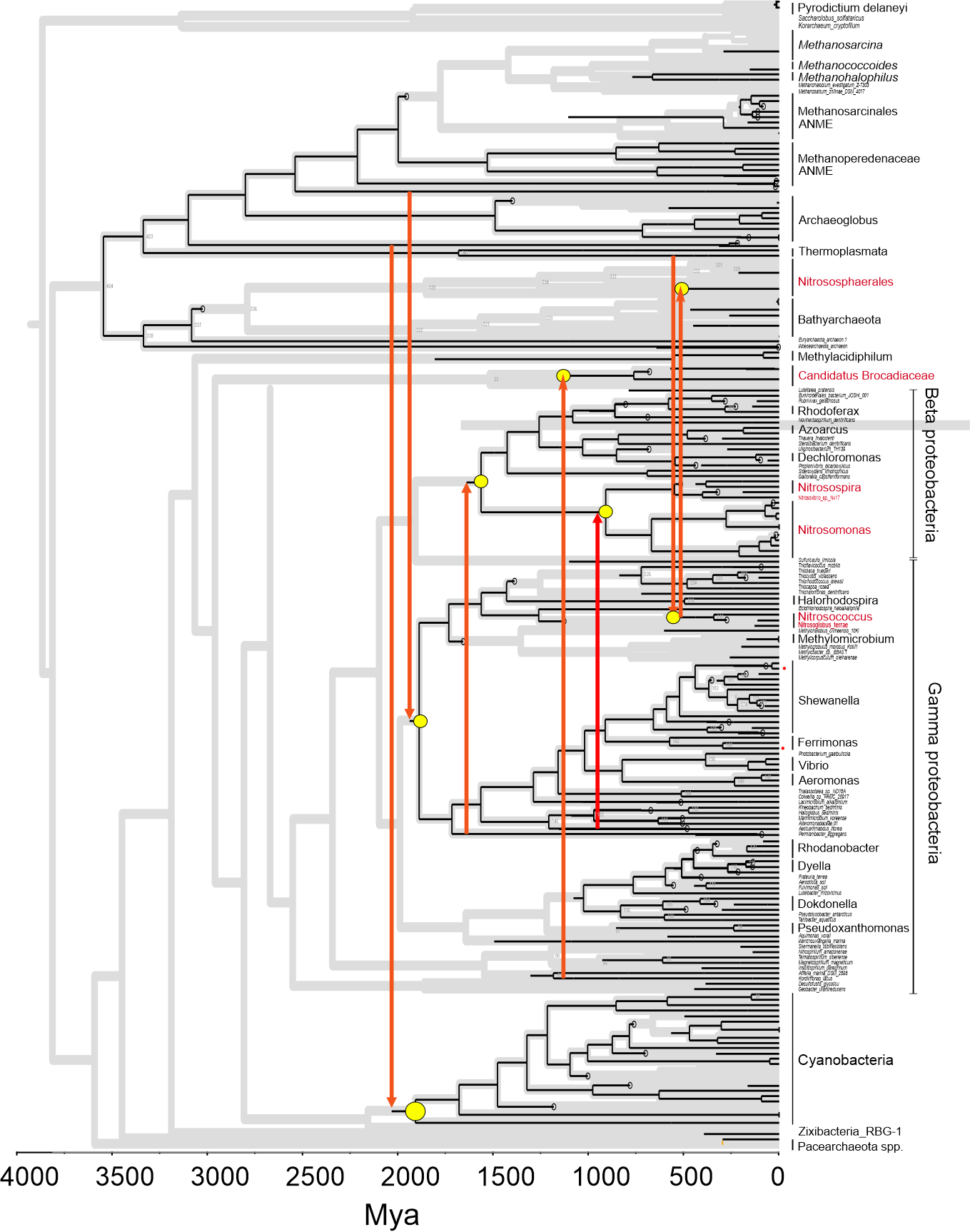


**Supplementary Figure 8** Visualized reconciliation of the electron transfer gene tree (MtrA) onto the dated species tree (grey) constructed from concatenated ribosomal genes. Black lines represent vertical inheritance within the genomes of the species tree, whereas red lines represent the selected gene transfer events for bacterial clades and ammonia-oxidizing bacteria (Red and black bold species-tree tips). HGT recipient nodes are shown with yellow circles.


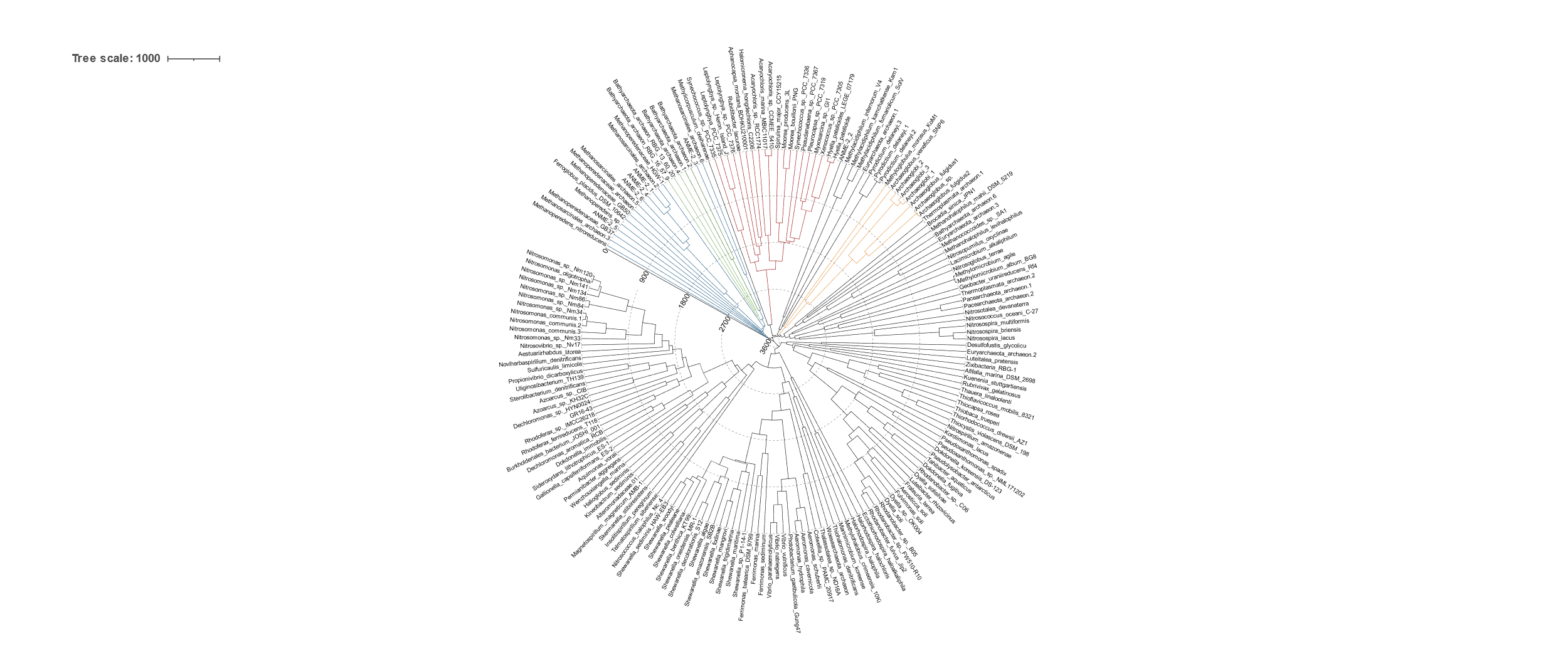


**Supplementary Figure 9** MtrA gene tree using the age estimates for the crown groups from the species tree as secondary calibrations

**Supplementary Table 6:** The single-copy genes searched in the proteomes of the species tree (48)


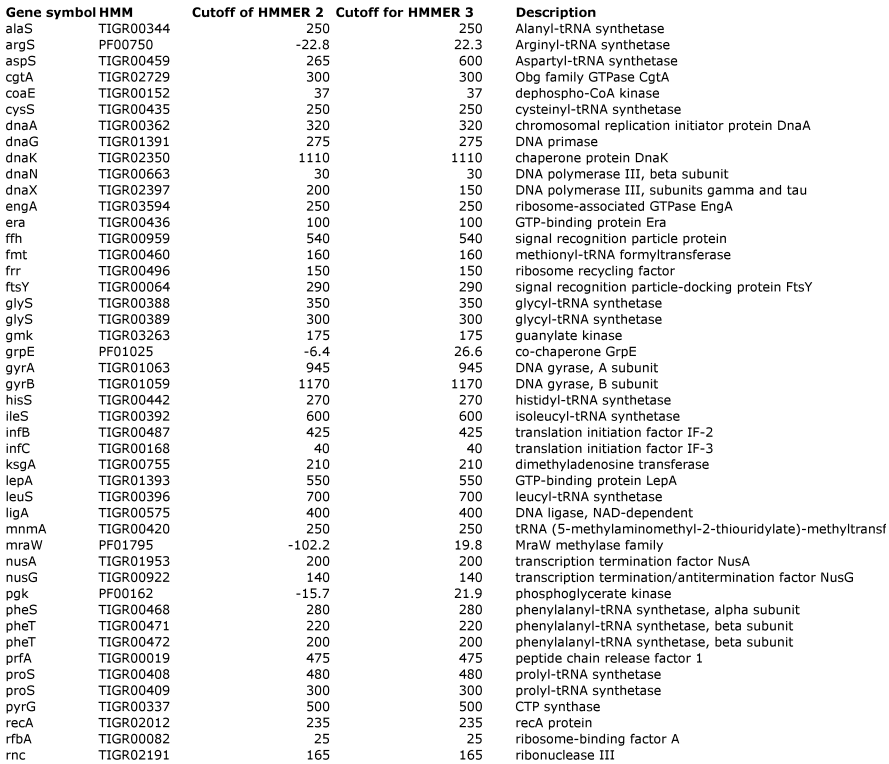


**Supplementary Table 6:** Continue (48)


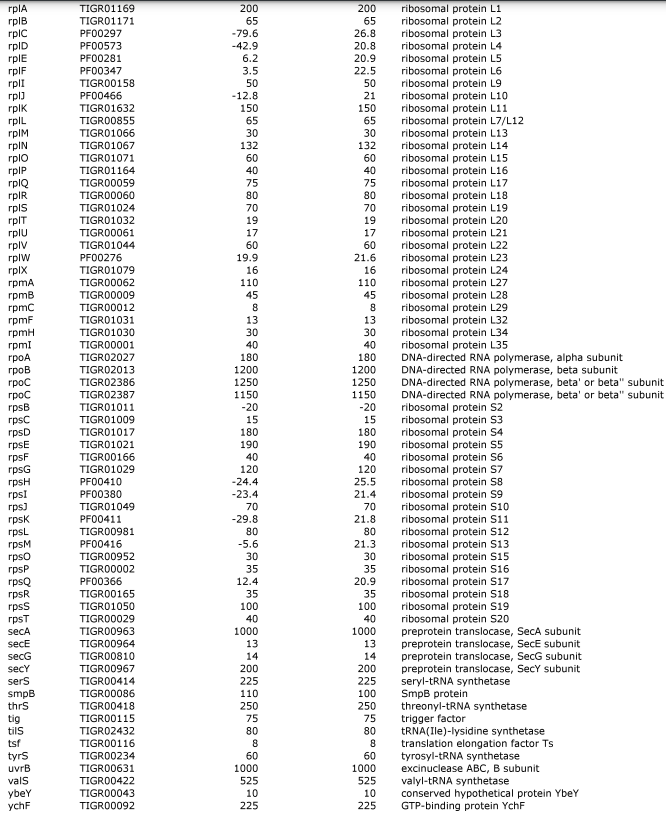


**Supplementary Table 7:** Calibrations used in molecular clock models. All calibrations are listed in Ma. Taxon 1 and Taxon 2 refer to the taxa used in PhyloBayes commands

**Supplementary Table 8:** The single-copy genes searched in the proteomes of the species tree(48)

| **CROWN GROUPS** | **Age range (both)** | | **Uni-CIR** | | **UNI-BD** | |
| --- | --- | --- | --- | --- | --- | --- |
|  | Min | Max | Min | Max | Min | Max |
| MethanoperedenaceaeANME | 2041 | 3008 | 2041 | 2898 | 2321 | 3008 |
| Archaeoglobus | 2013 | 3051 | 2337 | 3051 | 2013 | 3029 |
| Nitrosomonadecea | 1371 | 1973 | 1467 | 1973 | 1793 | 1371 |
| Rhodocyclales | 1414 | 2019 | 2019 | 1546 | 1838 | 1414 |
| Chromatiaceae | 1482 | 2337 | 2337 | 1847 | 2186 | 1482 |
| Shewanella | 1249 | 1686 | 1686 | 1276 | 1543 | 1249 |
| Vibrio | 465 | 869 | 869 | 474 | 818 | 465 |
| Aeromonas | 251 | 476 | 476 | 251 | 476 | 269 |
| Cyanobacteria | 2247 | 2722 | 2722 | 2247 | 2698 | 2372 |
